# The 37/67kDa laminin receptor (LR) inhibitor, NSC47924, affects 37/67kDa LR cell surface localization and interaction with the cellular prion protein

**DOI:** 10.1038/srep24457

**Published:** 2016-04-13

**Authors:** Daniela Sarnataro, Anna Pepe, Gennaro Altamura, Imma De Simone, Ada Pesapane, Lucio Nitsch, Nunzia Montuori, Antonio Lavecchia, Chiara Zurzolo

**Affiliations:** 1 Department of Molecular Medicine and Medical Biotechnologies, University of Naples “Federico II”, 80131, Naples, Italy; 2 Ceinge-Biotecnologie Avanzate scarl, 80145, Naples, Italy; 3 Department of Veterinary Medicine and Animal Productions, University of Naples “Federico II”, 80137, Naples, Italy; 4 Department of Translational Medical Sciences, University of Naples “Federico II”, 80131, Naples, Italy; 5 Department of Pharmacy, “Drug Discovery” Laboratory, University of Naples “Federico II”, 80131, Naples, Italy; 6 Institut Pasteur, Unité de Trafic Membranaire et Pathogénèse, 75724 Paris CEDEX 15, France

## Abstract

The 37/67 kDa laminin receptor (LR) is a non-integrin protein, which binds both laminin-1 of the extracellular matrix and prion proteins, that hold a central role in prion diseases. The 37/67 kDa LR has been identified as interactor for the prion protein (PrP^C^) and to be required for pathological PrP (PrP^Sc^) propagation in scrapie-infected neuronal cells, leading to the possibility that 37/67 kDa LR-PrP^C^ interaction is related to the pathogenesis of prion diseases. A relationship between 37/67 kDa LR and PrP^C^ in the presence of specific LR inhibitor compounds has not been investigated yet. We have characterized the trafficking of 37/67 kDa LR in both neuronal and non-neuronal cells, finding the receptor on the cell surface and nuclei, and identified the 67 kDa LR as the almost exclusive isoform interacting with PrP^C^. Here, we show that the treatment with the 37/67 kDa LR inhibitor, NSC47924, affects both the direct 37/67 kDa LR-PrP^C^ interaction *in vitro* and the formation of the immunocomplex in live cells, inducing a progressive internalization of 37/67 kDa LR and stabilization of PrP^C^ on the cell surface. These data reveal NSC47924 as a useful tool to regulate PrP^C^ and 37/67 kDa LR trafficking and degradation, representing a novel small molecule to be tested against prion diseases.

Several cell surface laminin-binding proteins have been described including integrin and non-integrin laminin receptors^1^. Among these, the 67 kDa LR (Laminin Receptor), which derives from a post-translational modification of its precursor, the 37 kDa LRP (Laminin Receptor Precursor), is a non-integrin protein with high affinity for laminin-1^2^. However, the precise molecular nature of 67 kDa LR is still controversial, since the biochemical changes, which accompany this alteration in molecular mass, are not yet known and there are a number of conflicting hypotheses. Among these, it has been suggested homodimerization, heterodimerization with an unidentified partner, or fatty acylation^3^,^4^,^5^.

The 37/67kDa laminin receptor, as cell surface receptor, has roles in cell migration, invasion, angiogenesis, extracellular matrix remodelling and apoptosis^6^,^7^,^8^,^9^,^10^,^11^,^12^,^13^,^14^. It was determined to be a highly conserved ribosomal protein that acquired its extracellular functions during evolution^15^. Such structural conservation implies that laminin receptor is important for basic cellular functioning, including its recently described role in telomerase activity^16^. Indeed, yeast homologs of 37/67 kDa LR are essential for cell viability, having roles in 20s–18 s rRNA processing and ribosome assembly^17^,^18^, as well as for cell signalling pathways that are important for cell survival^19^.

37/67 kDa laminin receptor is a multifunctional protein expressed within the cytoplasm, the nucleus and the plasma membrane of HeLa^2^,^20^,^21^ and BHK cells^22^; several isoforms of non-integrin laminin receptor are present in mouse brain^23^, the 67 kDa being the major form^24^.

The 37 kDa LRP has been identified as an interactor for the cellular prion protein PrP^C^ in a yeast two-hybrid screen^25^ and for prion-forming protein Sup35^26^; further *in vitro* studies in neuronal and non-neuronal cells, suggest that both the 37 kDa and 67 kDa LR isoforms could act as receptors for PrPs^27^,^28^. Furthermore, the laminin-1 binding domain on 37/67 kDa LR is identical to the PrP^C^ binding domain located between aa 161–180^25^,^29^.

PrP^C^ is an ubiquitous host protein expressed by all known mammals, whose function is not clear yet^30^. Its misfolded isoform PrP^Sc^ (scrapie isoform of PrP^C^) is the primary component of prions and is capable of seeding conformational conversion of PrP^C^ molecules in brains of humans or animals affected by prion diseases^31^,^32^. Recent studies showing colocalization of 37/67 kDa laminin receptor with PrP^CWD^ (Chronic Wasting Disease isoform of PrP^C^), ovine PrP^Sc^ and PrP^BSE^ (Bovine Spongiform Encephalopathy isoform of PrP^C^)^33^ together with the finding that bovine prions are endocytosed in an 37/67 kDa LR-dependent manner by human enterocytes^34^, confirm the relevance of non-integrin laminin receptor in the oral uptake of prions.

Interestingly, it has been previously shown that the 37 kDa LRP levels were increased in scrapie-infected murine N2a cells and in the brain and spleen of scrapie-infected mice, suggesting that LRP concentrations are correlated with PrP^Sc^ accumulation in organs from infected mice^25^. Moreover, the 37/67 kDa laminin receptor has been demonstrated to be required for PrP^Sc^ propagation in scrapie-infected neuronal cells^35^ leading to the possibility that PrP^C^-37/67 kDa LR interaction is related to pathogenesis of prion diseases.

Thereupon, several therapeutic approaches regarding prion diseases targeting 37/67 kDa LR have been developed^21^,^36^,^37^ including (a) anti-37/67 kDa LR antibodies^38^, (b) polysulfated glycans^39^, and (c) small interfering RNAs directed against the LRP mRNA^40^.

In the attempt to elucidate the nature of 37/67 kDa LR-PrP^C^ interaction and to evaluate, in different cell types, biological effects of a specific laminin receptor inhibitor, directed to the PrP^C^-laminin-1 active binding site of 37/67 kDa LR, we analyzed expression levels, intracellular distribution and 37/67 kDa LR -PrP^C^ interaction in neuronal and non-neuronal cells treated or not with the selective 37/67 kDa LR inhibitor NSC47924. This was the only compound, of previously identified virtual-screening forty-six molecules tested for laminin receptor inhibitory small molecules, which was able to selectively inhibit 37/67 kDa LR cell adhesion to laminin-1 in HEK-293 cells with IC50 and Ki values of 19.35 and 2.45 μmol/L^41^. However, a relationship between non-integrin laminin receptor and PrP^C^ in the presence of specific 37/67 kDa LR inhibitor compounds has not been investigated yet.

We report that both PrP^C^ and 37/67 kDa LR localized on the cell surface with predominance of the 37 kDa laminin receptor isoform on the plasma membrane. In addition, we found that the 67 kDa LR co-immunoprecipitated with PrP^C^ with a greater extent than the 37 kDa LRP, in both neuronal and non-neuronal cells.

Furthermore, the treatment with NSC47924 perturbs 37/67 kDa LR-PrP^C^ interaction both *in vitro* and in live cells impairing the immunocomplex formation through stabilization of PrP^C^ on the cell surface and induction of 37/67 kDa LR internalization, which progressively accumulates in early-endosome antigen 1 (EEA1)-enriched endosomes and subsequently in the endo-lysosomal compartment for its degradation.

## Results

### PrP^C^ and 37/67 kDa LR expression and intracellular localization in both neuronal and non-neuronal cells

**Figure 1:**
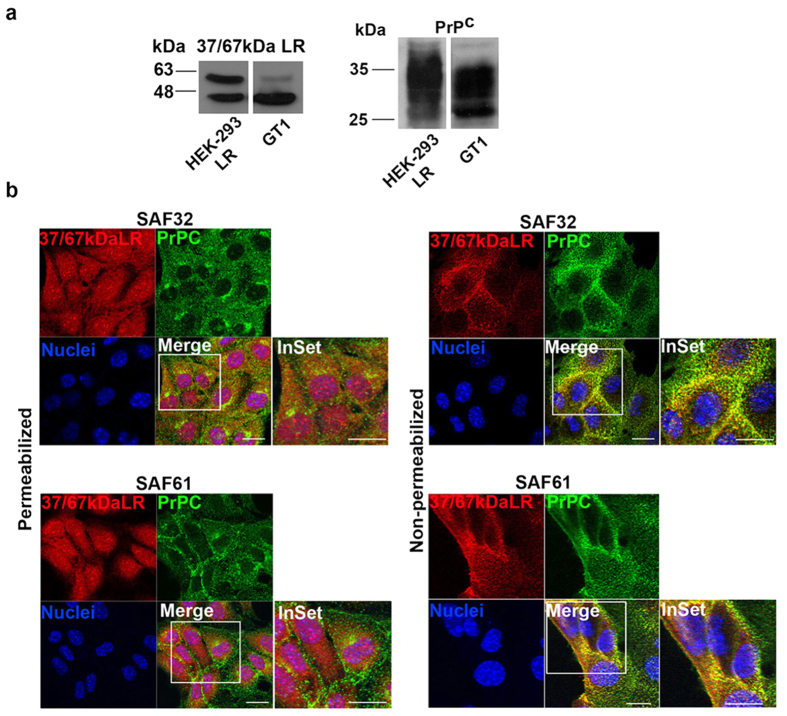
Expression levels and localization of 37/67 kDa LR and PrP^C^ in GT1 and HEK-293 cells. (**a**) GT1 and HEK-293 LR cells grown in DMEM supplemented with 10% foetal bovine serum, were scraped in lysis buffer and 80 μg of total proteins were subjected to SDS-PAGE. 37/67 kDa LR and PrP^C^ were revealed by Western blotting on nitrocellulose and hybridization with a 4290 pAb and SAF32 mAb, respectively. Molecular weights corresponding to the different bands revealed by ECL are indicated and expressed in kDa. (**b**) GT1 cells grown on coverslips and fixed in 4% paraformaldehyde, after 0.1% TX-100 permeabilization (left panel) or under non-permeabilized conditions (right panel), were double labelled with anti-PrP SAF32 (top panel) or SAF61 (bottom panel) antibody and 5004 anti-LR antibody; secondary Ab anti-mouse Alexa-488- (green) and anti-rabbit Alexa-546- (red) conjugated were used to reveal PrP^C^ and 37/67 kDa LR, respectively. Samples were observed using a laser scanning confocal microscope LSM 510 META Zeiss. InSet shows 2x zoom of images acquired with a 63x objective lens. Scale bar, 10 μm.

The endogenous laminin receptor from neuronal GT1 cell lysates migrated on SDS-PAGE as two different bands corresponding, respectively, to the laminin receptor precursor (37 kDa LRP) and the mature band (presumably the acylated isoform or the homodimeric/heterodimeric one) as shown before^2^. Lysates from HEK-293-LR cells stably transfected with a human 37LRP cDNA were loaded for reference^41^ (Fig. 1a). As already observed^3^, the precursor molecule showed an anomalous electrophoretic mobility, which accounts for an apparent molecular mass of ~44 kDa, and as previously described by Alqahtani *et al.*^42^, in cells expressing endogenous 37 kDa LRP and 67 kDa LR, the latter species runs in SDS–PAGE gels with an apparent molecular mass of ~60 kDa, which is lower than might be expected of a homodimer of 37 kDa LRP. The most likely explanation for this is that LR is resistant to denaturation in SDS, and thus maintains a more compact structure than during SDS–PAGE and runs with a higher electrophoretic mobility.

PrP^C^ was typically glycosylated, showing different bands ranging from ~27 to ~37 kDa; the unglycosylated form which migrates at ~27 kDa, the intermediate monoglycosylated form, which migrates at 28–30 kDa; and the highly glycosylated forms which migrate as bands spanning 33–37 kDa^43^,^44^. We next analyzed the subcellular localization of both laminin receptor and PrP^C^, by indirect immunofluorescence and confocal microscopy of neuronal GT1 cells grown on coverslips (Fig. 1b).

PrP^C^ was found mainly on the cell surface and in the Golgi area as previously demonstrated in GT1 cells and other neuronal cell lines^45^,^46^,^47^, while the receptor, accordingly to previous data showing a nuclear localization of laminin receptor and its interaction with histones^22^,^48^, showed a nuclear-like and plasma membrane staining pattern (Fig. 1b). Yellow-orange spots in the merged immunofluorescence images (Merge) of non-permeabilized cells in Fig. 1b (InSet), indicate that a fraction of 37/67 kDa LR is also found on the plasma membrane with PrP^C^.

The Pearson’s Correlation Coefficient PCC, produced a value of 0.78, which reflects the good degree of colocalization of the two proteins on the cell surface. We estimated, by Mander’s Colocalization Coefficient, MCC, that 72% of 37/67 kDa LR colocalized with PrP^C^ on the plasma membrane and that 45% of PrP^C^ colocalized with 37/67 kDa LR. These data indicate that the remaining 28% of laminin receptor could be free to be engaged by other interactors, such as laminin-1.

To assess the precise nature of receptor subcellular localization, 37/67 kDa LR co-immunostaining was performed also with the antibody specific for molecular markers of different intracellular organelles, such as Golgin (for Golgi apparatus), KDEL (for the ER), EEA1 (for early endosomes). Transferrin Alexa 488-conjugated (Tfr) was used to label recycling endosomes and Lysotracker red for lysosomes (see Supplementary Fig. S1). As expected, the PCC test produced a value close to zero, which indicated the absence of laminin receptor in the Golgi apparatus, as well as in other intracellular organelles.

### Interaction between 37/67 kDa laminin receptor and PrP^C^

*In vitro* identification of direct and heparan sulphate proteoglycan-dependent interaction sites mediating the binding of the cellular PrP to laminin receptor^49^ and the findings concerning the high-affinity binding curve between GST-PrP and iodinated laminin receptor^50^, prompted us to characterize the interaction between 37/67 kDa LR and PrP^C^ in mammalian neuronal and non neuronal cells.

**Figure 2:**
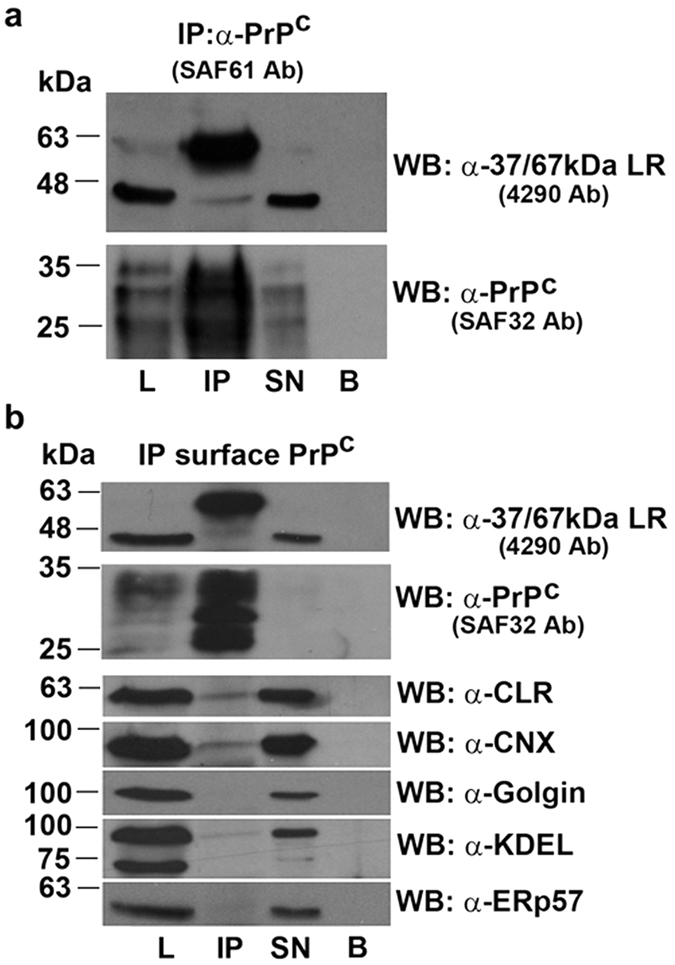
37/67 kDa LR and PrP^C^ co-immunoprecipitate on the surface of GT1 cells. (**a**) GT1 cells were grown on 150 mm dishes, lysed and PrP^C^ was immunoprecipitated using SAF61 mAb. 37/67 kDa LR was revealed using 4290 pAb. The membrane was stripped and blotted with SAF32 mAb to confirm the occurrence of the immunoprecipitation. L: input, IP: immunoprecipitated, SN: supernatant, B: protein-A beads alone. (**b**) GT1 cells grown on 150 mm dishes were first incubated with SAF61 mAb 1 hr at 4 °C, then washed and lysed in lysis buffer. PrP^C^ was immunoprecipitated with protein-A beads which were loaded on gels and transferred to nitrocellulose. Western blotting was performed by incubation with anti-37/67 kDa LR 4290 pAb. To confirm immunoprecipitation the membrane was stripped and probed with anti-PrP SAF32 mAb. Calnexin, Calreticulin, Golgin, KDEL and ERp57, were revealed by Western blotting with the specific antibodies on the same membranes after stripping. Note the presence of CLR and CNX, as well as the absence of Golgin, KDEL and ERp57 in the IP lane. L: input, IP: immunoprecipitated, SN: supernatant, B: protein-A beads alone. Anti-KDEL antibody detects two bands of ~94 kDa and ~78 kDa.

In order to understand whether PrP^C^ and 37/67 kDa LR entertained physical interaction in the cells, we subjected them to co-immunoprecipitation (Co-IP) from total cell lysates. Specifically, we first immunoprecipitated PrP^C^ from lysates and then immuno-identified laminin receptor in the precipitate by Western blotting. As shown in the Fig. 2a, the 67 kDa isoform of laminin receptor could be immunoprecipitated with a greater extent (compared to immature 37 kDa LRP) along with PrP^C^ in GT1, as well as in HEK-293 cells (see Supplementary Fig. S3), whereas the precursor remained in major measure in the supernatant (SN) of the immunoprecipitate. Importantly, to confirm the occurrence of the co-immunoprecipitation, we used an anti-laminin receptor antibody in the precipitation step and one against PrP^C^ to visualize prion protein in the Western blotting of the immunoprecipitate (see Supplementary Fig. S2).

To specifically discern the cellular compartment involved in 37/67 kDa LR-PrP^C^ interaction, we performed cell-surface co-immunoprecipitation assays in the same conditions of the previous immunoprecipitation experiment with the exception that here (Fig. 2b) the cells were incubated on ice with the anti-PrP antibody SAF61, before lysis. As expected from the immunofluorescence data, the two proteins were both present at the cell surface, and again almost exclusively the mature 67 kDa LR co-immunoprecipitated with PrP^C^ on the surface of live cells. Calnexin and Calreticulin (ER chaperones markers, CNX and CLR), which were used as control of the specificity of the immunoprecipitation step, were also detectable in the immunocomplex. The presence of Calnexin and Calreticulin in the IP against PrP^C^ from the cell surface was unexpected and is further discussed below (see Discussion). Moreover, we conceive that cell surface PrP^C^ is complexed with cell surface CRL and CNX, but that this doesn’t show up in whole cell immunoprecipitates, because during the cell surface binding of PrP^C^ with SAF61 antibody we selectively enrich the CNX and CLR complexed with PrP^C^ on the plasma membrane.

Therefore, in order to ascertain the specificity of the Co-IP between LR and PrP^C^, the same membranes used for immunoblotting with anti-CNX and anti-CLR antibodies, were stripped and hybridized with anti-Golgin antibody, a marker of the Golgi apparatus and anti-KDEL and ERp57 antibodies to test the presence of other Endoplasmic Reticulum-(ER) proteins in the IP. Protein-A beads alone were carried as control of the assay (Fig. 2b, lane B). Notably, as shown in the Fig. 2b, Golgin, as well as KDEL and ERp57 proteins were absent from the PrP^C^-37/67 kDa LR immunocomplex.

### The 37/67 kDa laminin receptor inhibitor NSC47924 impairs PrP^C^-37/67 kDa LR binding both *in vitro* and in live cells

**Figure 3:**
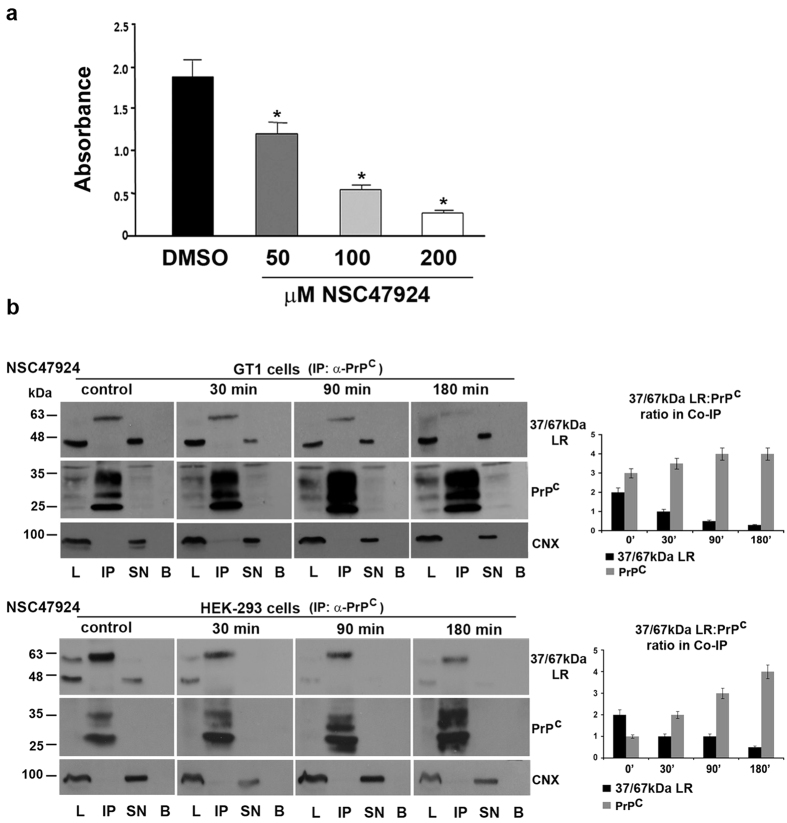
37/67 kDa LR inhibitor NSC47924 impairs recombinant PrP-r37LRP binding *in vitro* and perturbs co-immunoprecipitation of 37/67 kDa LR and PrP^C^ in live cells. (**a**) Purified human His-tagged recombinant 37LRP (r37LRP) was placed for 1 hour at 37 °C on wells coated with 125 ng of human recombinant PrP, in the presence of 50, 100 and 200 μM NSC47924, or DMSO as a vehicle control. Bound r37LRP was revealed by anti-His-HRP and OPD staining; the absorbance at 490 nm was measured. r37LRP binding to BSA-coated wells was subtracted to obtain specific binding. Values represent the mean ± SEM of three experiments carried out in triplicate; (^*^P < 0.05). (**b**) Co-IP in GT1 and HEK-293-LR cells was carried out after inhibitor treatment in kinetic assays, for indicated times, using anti-PrP SAF32 mAb to immunoprecipiate PrP^C^ and anti-37/67 kDa LR 4290 pAb to reveal 37/67 kDa LR in the IP. Immunoblotting with SAF32 mAb was performed to ascertain the occurrence of IP. Calnexin was revealed by stripping the same membranes used above, immunoblotted with anti-CNX antibody and revealed with ECL. For comparison, signals of CNX are shown to exclude both aspecifics and defect in loading samples on gels. 37/67 kDa LR/PrP^C^ ratio in the immunocomplex was determined by imposing as 100% the sum of 37/67 kDa LR and PrP^C^ signals in the IP. To calculate ratio, densitometric analysis was performed with ImageJ software and values from the mean ± SEM of three experiments, were considered (*P* < 0.05). All data were statistically significant.

Previous studies demonstrated the requirement of the 37/67 kDa LR system for prion propagation *in vitro*^27^,^35^. Thus, a possible anti-prion therapy approach could target the 37/67 kDa LR to impair 37/67 kDa LR-PrP^C^ interaction. To this aim, we decided to study whether NSC47924, an inhibitor of 37/67 kDa LR binding to laminin-1^41^, and directed to a laminin-active site of 37/67 kDa LR, which is shared with PrP^C^, influenced the interaction between 37/67 kDa LR and PrP^C^ and eventually their intracellular trafficking. NSC47924 is directed to the “peptide G” domain of 37 kDa LRP (aa 161–180), which is also the domain of HSPG-independent binding of 37/67 kDa LR to PrP^C^^49^. Since 67 kDa LR derives from homo or hetero-dimerization of 37 kDa LRP^3^,^4^, NSC47924 is likely an inhibitor of both isoforms. Therefore, the ability of NSC47924 to inhibit the binding of human recombinant soluble 37LRP (r37LRP) to human recombinant PrP was first evaluated by ELISA assays (Fig. 3a). Purified His-tagged r37LRP was incubated on wells pre-coated with human recombinant PrP, and binding was detected by anti-His HRP. As a control for binding specificity, r37LRP binding to BSA-coated wells was also evaluated in parallel and the absorbance readings subtracted. r37LRP binding to recombinant PrP was specifically inhibited by NSC47924. Thus, NSC47924 is a specific inhibitor of 37/67 kDa LR direct binding to PrP^C^, *in vitro*.

To test the compound in live cells, we performed co-immunoprecipitation assays in both control condition and treatment of GT1 and HEK-293 cells with the inhibitor (20 μM NSC47924) (IC50 = 20 μM for inhibition of 37/67 kDa LR binding to laminin-1)^41^. Interestingly, the presence of NSC47924 in kinetic Co-IP assays (Fig. 3b), impaired the formation of the 37/67 kDa LR-PrP^C^ immunocomplex in GT1, as well as in HEK-293 cells, in a time-dependent manner; furthermore, inhibitor incubation induced a decrease of 67 kDa LR and a progressive and substantial increase of PrP^C^ in the immunocomplex (Fig. 3b, IP lane), as shown by the protein ratios plotted in the graphs (Fig. 3b, right panels).

### The NSC47924 inhibitor affects the trafficking of 37/67 kDa LR from the cell surface stabilizing PrP^C^ on the plasma membrane

To understand why NSC47924 exerts the aforementioned effects and dissect the mechanism by which it acts in the cells, we decided to investigate whether the localization and intracellular trafficking of 37/67 kDa LR and PrP^C^ were perturbed by the inhibitor treatment.

**Figure 4:**
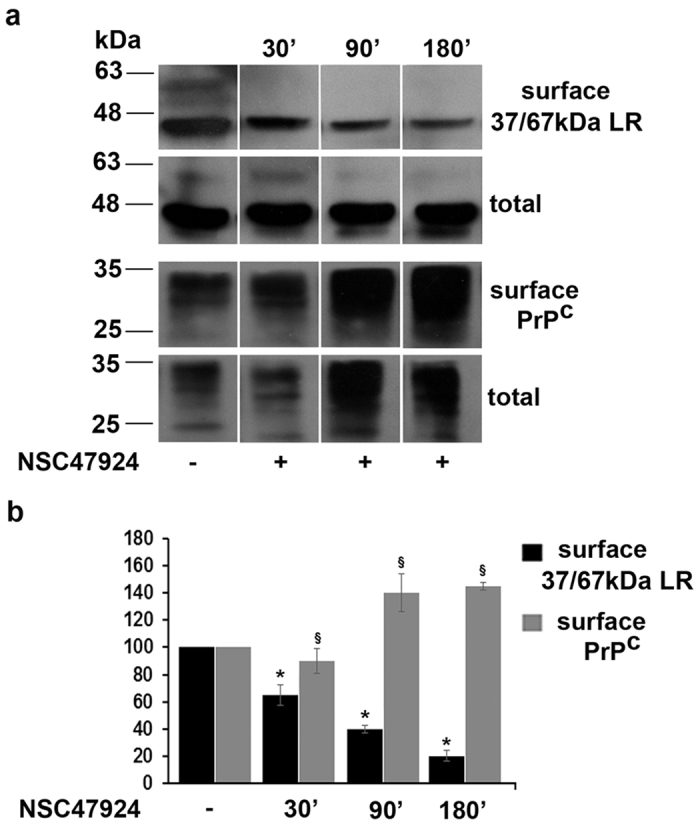
The inhibitor NSC47924 interferes with plasma membrane localization of 37/67 kDa LR. (**a**) GT1 cell surface proteins were biotinylated at 4 °C in control conditions (without inhibitor, -) or after 30 min, 90 min and 180 min of treatment with NSC47924 at 37 °C, and were recovered from cell lysates by immunoprecipitation with streptavidin-beads. Total (80 μg of total cell lysates) and cell surface proteins (IP from streptavidin beads), were loaded on gel and processed for SDS-PAGE and ECL. 37/67 kDa LR and PrP^C^ were immunodetected by blotting with 4290 pAb and SAF32 mAb, respectively. (**b**) The plot shows the relative percentage of 37/67 kDa LR and PrP^C^ on the surface of the cells after indicated times of NSC47924 treatment, using as 100% the maximal expression value at cell surface in control conditions (NSC47924 -). Data are expressed as the means ± SEM of three independent experiments (^*,§^*P* < 0.05).

Thus, we performed kinetic biotinylation assays (Fig. 4a) in which, GT1 cell surface proteins were biotinylated before and after inhibitor treatment at different times. 37/67 kDa LR and PrP^C^ were immunoidentified with the specific primary antibodies by a Western blot analysis on both streptavidin-immunoprecipitated materials (which contains only biotinylated proteins) and total cell lysates (which contains biotinylated and non-biotinylated proteins). We found that the 37 kDa LRP was the most represented isoform both on the plasma membrane and in the total cell lysates. This latter isoform together with the 67 kDa LR, starting from 30 min up to 180 min of NSC47924 treatment, progressively decreased from the cell surface in a time-dependent manner (Fig. 4b), possibly due to a stimulated internalization and degradation. Interestingly, the presence of NSC47924 induced stabilization of cell surface PrP^C^ (Fig. 4a, bottom panel), thus reinforcing the results found in the Co-IP assays, where the presence of 67kDa LR in the immunocomplex decreased in favour of PrP^C^ (see Fig. 3b).

In parallel, we employed double indirect immunofluorescence kinetic assays monitoring the presence of both 37/67 kDa LR and PrP^C^ on the cell surface after different times of NSC47924 treatment under non-permeabilized conditions. To this aim, GT1 cells were treated or not (control) with the inhibitor for the same times of the biotinylation assays; after that, as shown in Supplementary Fig. S4, the cells were fixed and processed for immunofluorescence to analyse the presence of 37/67 kDa LR and PrP^C^ on the cell surface. In agreement with biotinylation experiments, we found that while cell surface signal of 37/67 kDa LR decreased on the plasma membrane in a time-dependent manner, PrP^C^ was stably present on the cell surface indicating an opposite effect of the compound on the trafficking of the two proteins.

**Figure 5:**
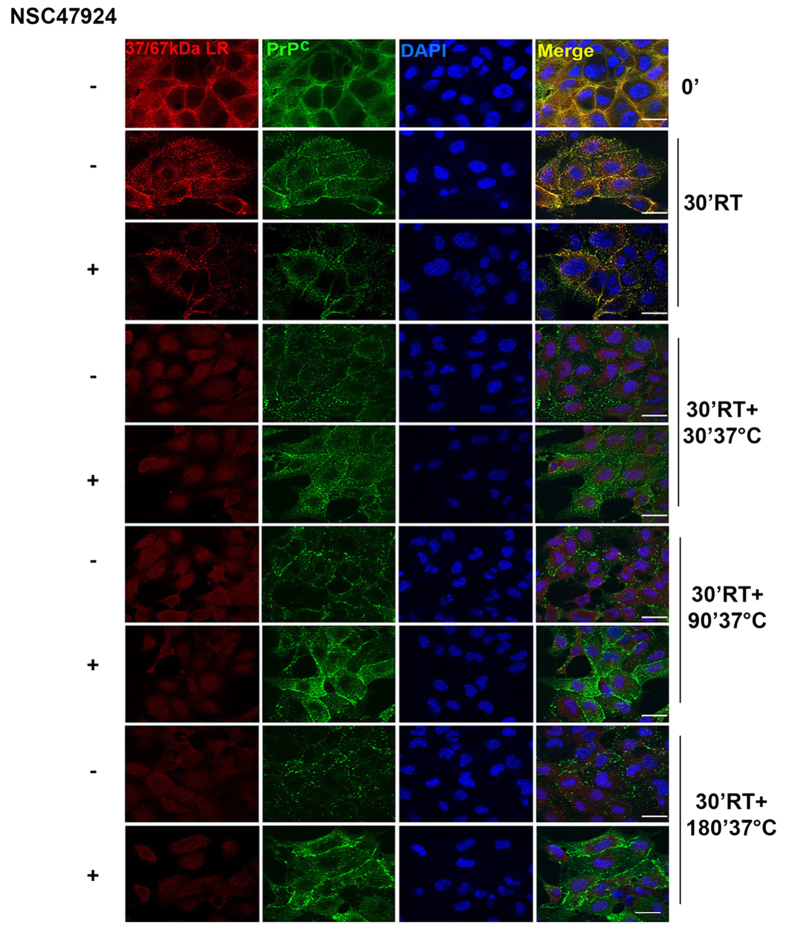
NSC47924 affects the intracellular fate of 37/67 kDa LR and PrP^C^. GT1 cells were grown on coverslips, cooled at 4 °C on ice, and stained with SAF32 mAb (1:100) and 5004 pAb (1:50) to label cell surface PrP^C^ and 37/67 kDa LR, respectively. To allow membrane trafficking and internalization, GT1 cells were warmed at 37 °C in the culture medium with or without NSC47924 for indicated times. After that, the cells were washed, fixed in PFA 4% and permeabilized in 0.1% TX-100 for 10 min. To detect internalized proteins the cells were labelled with secondary antibodies (PrP^C^, green; 37/67 kDa LR, red) and their colocalization was measured. RT: Room Temperature; Scale bars, 10 μm.

In order to specifically investigate the effect of NSC47924 on the intracellular fate of the two proteins, we performed double indirect immunofluorescence assays under permeabilized conditions, following specifically the cell surface laminin receptor and PrP^C^ pools (Fig. 5). GT1 cells, were first incubated on ice (t = 0′ at 4 °C, which blocks membrane trafficking)^51^ with the primary antibodies against both laminin receptor and PrP^C^ and then treated at 37 °C (which allows membrane trafficking) with or without the inhibitor, from 30 min up to 180 min, fixed and permeabilized. Accordingly to Co-IP assays, the two proteins colocalized on the cell surface and already after 30 min at room temperature (RT), both 37/67 kDa LR and PrP^C^ entered the cells, as shown by the punctate staining. The presence of the inhibitor, (t = 30′ RT, room temperature, + NSC47924) induced internalization of 37/67 kDa LR and a decrement of PrP^C^ colocalization, which was totally abolished with increasing times of inhibitor treatment (t = 90′ and 180′). The disappearance of punctate laminin receptor staining which resulted somewhat sparse into the cells and sharply less intense compared to control, supports the results from biotinylation kinetics (Fig. 4), confirming that the inhibitor stimulates 37/67 kDa LR internalization and possibly its degradation, while PrP^C^ was stabilized on the cell surface due to its prevented internalization. This effect could be explained by assuming that PrP^C^ becomes not able to be internalized because of the absence of its receptor.

**Figure 6:**
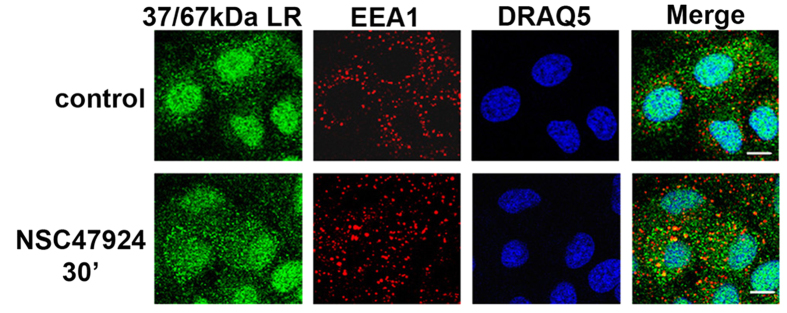
The inhibitor NSC47924 induces 37/67 kDa LR internalization via early endosomes. GT1 cells grown on coverslips in both control (without inhibitor) and treated conditions (30 min NSC47924) were fixed, permeabilized with TX-100 and probed with 5004 pAb and anti-EEA1 mAb to label 37/67 kDa LR and early endosomes, respectively. Secondary antibodies Alexa-488- and Alexa-546-conjugated were used to visualize 37/67 kDa LR and EEA1. Nuclei were stained with DRAQ5 dye (blue). Distribution of 37/67 kDa LR and EEA1 were analyzed by confocal microscopy. Scale bar, 10 μm.

**Figure 7:**
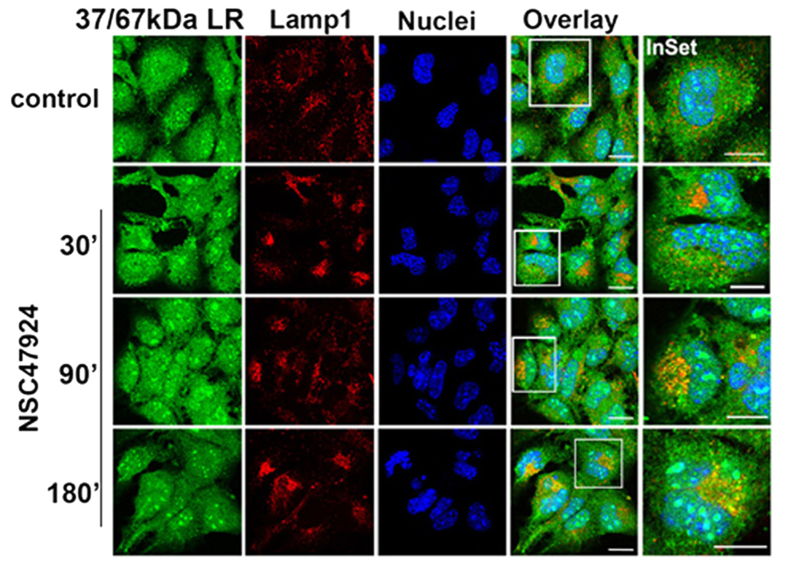
The inhibitor NSC47924 induces a time-dependent accumulation of 37/67 kDa LR in endo-lysosomes. GT1 cells grown as above (see Fig. 6) were treated with NSC47924 for indicated times and immunostained with anti-37/67 kDa LR 5004 antibody (green) and anti-Lamp1 (marker of endo-lysosomes, red). Distribution of 37/67 kDa LR and Lamp1 were analyzed by confocal microscopy. Scale bar, 10 μm.

To characterize 37/67 kDa LR internalization in GT1 cells, we performed double immunofluorescence assays by labelling laminin receptor and a marker of early endosomal pathway EEA1, followed by confocal microscopy analysis (Fig. 6). Differently from untreated GT1 cells, 37/67 kDa LR colocalized with EEA1 under NSC47924 treatment. A PCC average value higher than 0.6 (N = 100) was found. This partial early endosomal accumulation was clearly evident after 30 min inhibitor treatment. Interestingly, NSC47924 treatment induced a substantial re-localization of 37/67 kDa LR in the endo-lysosomal compartment, as indicated by colocalization with Lamp-1 in double indirect immunofluorescence assays (Fig. 7). A PCC average value higher than 0.7 (N = 100) was found after 90 min and a value of 0.8 after 180 min. We confirmed 37/67 kDa LR internalization via early-endosomes (Fig. 6) and its lysosomal-mediated degradation (Fig. 7 and Supplementary Fig. S5). Indeed, the effect of inhibiting lysosomal protein degradation on 37/67 kDa LR fragment generation, was determined incubating GT1 cells for 2 days in culture medium containing 20 mM NH4Cl, in the presence (30′, 180′) or not (−) of NSC47924 inhibitor (Supplementary Fig. S5c,d). We found that NH4Cl treatment resulted in accumulation of 37/67 kDa LR and inhibition of 37/67 kDa LR fragment generation (180′ NSC47924 control *versus* + NH4Cl, and quantification in panel d) implying that NSC47924 also affects 37/67 kDa LR lysosomal-mediated degradation. Moreover, as shown in Fig. S6, the fragment did not reach the cell surface.

Taken together, these results indicate that the inhibitor specifically interferes with 37/67 kDa LR cell surface interaction with PrP^C^, stimulating internalization of both 37 kDa and 67 kDa LR isoforms suggesting that NSC47924 might act through sterical hindrance contrasting physiological 37/67 kDa LR cell surface interaction with laminin-1 and PrP^C^.

## Discussion

Mapping analysis in yeast two-hybrid system and cell-binding assay identified PrPLRPbd1 (amminoacids aa 144–179) as a direct and PrPLRbd2 (amminoacids aa 53–93) as an indirect HSPG-dependent laminin receptor precursor (LRP)-binding site on PrP^C^^49^.

Furthermore, cell-binding and internalization studies proved that 37/67 kDa LR acts as the receptor for PrPs on the cell surface^39^. Here, we analysed the interaction between the non-integrin laminin receptor and cellular PrP by screening for the binding of all LR isoforms with PrP^C^ by co-immunoprecipitation assays, in both neuronal and non-neuronal cell systems. Despite previous and controversial findings of interaction between 37 kDa LRP and FLAG-PrP in transiently co-transfected COS-7 cells^25^, the localization of this interaction and its physiopathological role are still debated, therefore our attempt to investigate the interaction between the two proteins under physiological conditions in neuronal cells is novel.

In particular, we asked whether the two proteins interacted in neuronal cells, as well as in non-neuronal cells, and which of the laminin receptor isoforms specifically interacted with PrP^C^.

Since PrP^C^ is localized on the surface of neuronal cells^52^,^53^,^54^ and 37/67 kDa LR is also present in neuronal cells^55^, we hypothesized that the interaction between the two proteins could occur on the surface of the cells. By double immunofluorescence assay in non permeabilized cells and a cell-surface immunoprecipitation of PrP^C^ followed by detection of laminin receptor by immunoblotting, we could show that the two proteins colocalized on the plasma membrane and that PrP^C^ preferentially co-immunoprepitated with the mature 67 kDa LR.

Interestingly, we detected the presence of Calnexin and Calreticulin, which are typical molecular chaperones resident in the ER, in the surface immunoprecipitate. This finding was (Fig. 2b, bottom panels) unpredicted regarding the co-immunoprecipitation with PrP^C^, but was in agreement with other results in which it has been demonstrated that typical ER proteins, like Calreticulin, could be located on the cell surface also^56^,^57^. The marked co-immunoprecipitation of PrP^C^ with Calnexin and Calreticulin on the plasma membrane is particularly interesting considering that catalysis of protein folding is controlled by the interaction with these two chaperones^58^ and that PrP^C^ to PrP^Sc^ conversion is underlined by unfolding process^43^. Moreover, the presence of ER chaperones CNX and CLR (which retains a KDEL signal), in the surface Co-IP materials, was specifically confirmed by the absence of Golgi resident proteins (as Golgin), as well as the absence of other ER-resident proteins, such as ERp57 (which often associates with CNX in the ER), and other KDEL proteins. Accordingly to previous results in neuronal cells^57^, where a significant fraction of CNX was found on the cell surface of neurons and that ERp57, in contrast to CNX, was highly enriched in the non-synaptic membrane fractions, our findings indicate that CNX and ERp57 not necessarily follow the same fate into the cells.

Moreover, although CLR is a KDEL protein, we found only a negligible KDEL band in the Co-IP material. The most likely explanation for this is that the complex of CLR with cell surface PrP^C^ could somehow mask the KDEL signal, rendering CLR “not well recognizable” by the anti-KDEL antibody. Therefore, Co-IP assays from the cell surface give rise to a series of questions, among these, whether PrP^C^ on the cell surface of GT1 cells is properly folded or not. However, this phenomenon deserves further investigation.

Although the interaction between 37/67 kDa LR and PrP^C^ has been previously proposed, our results provide the first clear evidence that this interaction occurs on the plasma membrane, suggesting that the 67 kDa LR may act as a PrP^C^ cell surface receptor in neuronal mammalian cells.

To analyze the nature of 37/67 kDa LR-PrP^C^ interaction, we decided to study whether the laminin recepotor inhibitor NSC47924, targeting the peptide G sequence of 37/67 kDa LR (residues 161–180) able to directly bind both laminin-1^29^ and PrP^C^^49^, influenced the interaction between 37/67 kDa LR and PrP^C^ and eventually their intracellular trafficking. NSC47924 specifically inhibited 37/67 kDa LR direct binding to PrP^C^ in ELISA assays and perturbed the interaction between the two proteins in live cells also. We speculate that is most likely because NSC47924 is able to antagonize both the direct, peptide G-mediated, 37/67 kDa LR binding to PrP^C^ and the HPSGs-mediated indirect one, which occurs in live cells^49^.

The drug treatment induces cell surface disappearance of 37/67 kDa LR and accumulation in early and late endosomes/lysosomes, stabilizing cell surface distribution of PrP^C^, suggesting that the inhibitor impairs 37/67 kDa LR trafficking and definitively interaction with PrP^C^.

We envisage the following hypothesis to explain our findings: the inhibitor NSC47924 could exploit its effect on the cell surface 37/67 kDa LR by perturbing receptor anchorage to both laminin-1 and PrP^C^, thus rendering the 37/67 kDa LR more prone to be internalized. It is likely that this compound could exert its effect on both bound and free 37/67 kDa LR on the cell surface, inducing its internalization and degradation. However, once internalized the 37/67 kDa LR partially accumulates in EEA1 enriched-endosomes and, ultimately, into endo-lysosomal vesicles. This effect dramatically decreases cell binding to the laminin of extracellular matrix^41^, for which peptide G is crucial^29^. Thus, the fact that the presence of 37/67 kDa LR decreased along with increasing times of treatment, resulting in its partial early endosomes accumulation and progressive late endo-lysosomal localization after inhibitor incubation, suggests that the intracellular trafficking and presumably the normal endocytic pathway of 37/67 kDa LR is altered by NSC47924. This compound, inducing 37/67 kDa LR internalization, is able to subtract laminin receptor from binding to PrP^C^, which in presence of the inhibitor, is no longer able to be internalized.

These findings, on one hand, are very crucial if we consider that the main function of 37/67 kDa LR is to enhance tumor cell adhesion to the laminin of basement membranes and cell migration, two key events in the metastasis cascade^2^. Thus, inhibiting 37/67 kDa LR binding to laminin^8^,^9^,^10^,^11^, as well as modulating its expression on the cell surface and degradation could be a feasible approach to block tumor invasion.

On the other hand, the effect that the 37/67 kDa LR inhibitor exerts on PrP^C^, stabilizing its cell surface localization, provides good hopes to test it against prion conversion, which has been demonstrated to occur on the plasma membrane^59^ and/or intracellular compartments^60^. However, whether the effects we observed, due to the inhibitor, could result in a stimulus or a stumbling block to determine PrP^C^ to PrP^Sc^ conformational transition has to be established with further investigations.

Moreover, the finding that 37/67 kDa LR may play a role in Alzheimer’s disease (AD)^61^,^62^,^63^ and that modulation of 37/67 kDa LR could affect Aβ cytotoxicity^64^,^65^ and its release from cells^66^, highlights the importance of NSC47924 inhibitor to modulate 37/67 kDa LR trafficking and degradation, and the consequences that this inhibitor could have on other neurodegenerative disorders.

## Methods

### Reagents and antibodies

Cell culture reagents were purchased from Gibco Laboratories (Grand Island, NY). The SAF32 and SAF61 antibodies (directed, respectively, against the N- and C-terminal domain of PrP^C^) were from Cayman Chemical (USA). Production of recombinant and anti-67LR antibodies: a recombinant His-tagged 37LRP polypeptide (r37LRP) was made in bacteria and Nickel affinity purified, as described^41^,^67^. The polyclonal 5004 Ab able to recognize both the 37 kDa and the 67 kDa forms of LR by immunofluorescence, Western blot analysis and immunoprecipitation was made against a recombinant 37 LRP^67^, the polyclonal 4290 Ab was made against a C-terminal peptide derived from LR^29^ and was a kind gift from Dr. Mark E. Sobel (Bethesda, MD); the NSC47924 inhibitor has been already described^41^. (Human) Recombinant PrP (PO2) was from Abnova (Taipei, Taiwan). Protein-A-Sepharose was from Pharmacia Diagnostics AB (Uppsala, Sweden). Transferrin Alexa-488-conjugated (Tfr488), Alexa-488-, Alexa-546-conjugated secondary Abs and Lysotracker Red DND-99 were from Invitrogen (Molecular Probes). The anti- KDEL, anti-CNX, anti-CLR, anti-Golgin, anti-EEA1 and anti-ERp57 antibodies were from StressGen Biotechnologies Corp (Victoria, BC, Canada). Anti-Lamp1 antibody was from BD Pharmigen and anti-β-tubulin antibody was from Abcam. DRAQ5 and DAPI dyes were purchased from Cell Signal Technology. Biotin-LC was from Pierce and all other reagents were obtained from Sigma Chemical Co. (St Louis, MO).

### Cell culture and drug treatment

GT1 (hypothalamic neuronal mouse cell line) and HEK-293-LR (HEK-293 stable expressing T7/His-tagged 37LRP)^41^ were grown in Dulbecco’s modified Eagle’s medium (DMEM), with 4500 mg/glucose/L, 110 mg sodium pyruvate and L-glutamine (SIGMA D6429), supplemented with 10% fetal bovine serum. Cells were cultured at 37 °C under 5% CO_2_.

For inhibitor NSC47924 treatment, the cells were washed in serum free medium, incubated for 30 min at room temperature (RT) in Areal medium (13.5 g/l of Dulbecco’s modified eagle’s medium with glutamine without NaHCO_3_ (SIGMA-D-7777), 0.2% BSA and 20 mM HEPES, final pH 7.5) and for further indicated times at 37 °C under 5% CO_2_ in the presence of 20 μM inhibitor in DMEM supplemented with 1% serum. NH4Cl (20 mM in culture medium) was used to inhibit endo/lysosomal protein degradation for 2 days.

### Indirect immunofluorescence and confocal microscopy

To analyze protein steady-state localization, GT1 cells were cultured to 50–70% confluence in growth medium for three days on coverslips, washed in PBS, fixed in 4% paraformaldehyde (PFA), permeabilized or not with 0.1% TX-100 for 10 min (where indicated) and processed for indirect immunofluorescence using specific antibodies 30 min in PBS/BSA 0.1%. The cells were incubated with anti-PrP SAF32 (IgG2) monoclonal antibody (2 μg/ml, 1:100) and anti-37/67 kDa LR rabbit 5004 policlonal antibody (1 μg/ml, 1:50) to label PrP^C^ and 37 kDa/67 kDa LR, followed by incubation with fluorophore-conjugated secondary antibodies. For lysosome staining, cells were incubated for 1 hr with Lysotracker (1:10000) in complete medium before fixing. Where indicated, anti-Lamp1 antibody was used as marker of endo-lysosmal compartment. For Tfr-Alexa 488 staining, the cells were incubated 45 min in complete medium before proceeding with immunofluorescence. Nuclei were stained by using DRAQ5 dye (1:5000) or DAPI (1:1000) in PBS.

To analyze cell surface localization of 37/67 kDa LR and PrP^C^, the cells were first incubated (or not, control) with the inhibitor for the indicated times and then fixed in PFA 4% and processed for immunofluorescence under non permeabilized conditions by labelling laminin receptor and PrP^C^ with the specific antibodies.

#### Internalization assay

In order to exclusively label the cell surface 37/67 kDa LR and PrP^C^ protein pools and to follow their fate from the cell surface, GT1 cells grown on coverslips were first incubated (pulse) with 5004 and SAF32 primary antibodies for 30 min at 4 °C (which blocks membrane trafficking events)^51^. Primary antibodies were diluted in Areal Medium. The cells were then washed to eliminate excess of unbound antibodies and then shifted at 37 °C under 5% CO_2_, to allow endocytic processes, for indicated times (chase) in the presence or not of 20 μM NSC47924 in the culture medium. GT1 cells were fixed and permeabilized before incubation with fluorescently-conjugated secondary antibodies and laser scanning confocal analyses^68^.

Pearson’s correlation coefficient (PCC) was employed to quantify colocalization^69^ between 37/67 kDa LR and PrP^C^ (as well as other intracellular markers), and was determined in at least 25 cells from four different experiments. PCC was calculated in regions of 37/67 kDa LR and reference protein co-presence^69^. In brief, the Otsu algorithm was applied to segment laminin receptor and PrP^C^ (as well as other intracellular markers) images, in order to define co-localization regions of the reference proteins. The PCC was then calculated in the defined regions for the images of interest. Whereas PCC provides an effective statistic for measuring overall association of two probes in an image, it has the major shortcoming that it indirectly measures the quantity of one protein that colocalizes with a second protein^70^. This quantity can be measured via Manders’ Colocalization Coefficient (MCC). The degree of colocalization between 37/67 kDa LR and PrP^C^ was quantified using the colocalization finder and JaCoP plug-in of ImageJ software (http://rsb.info.nih.gov/ij/). MCCs were measured for at least 25 cells per sample.

Immunofluorescences were analyzed by the confocal microscope Zeiss META 510 equipped with an oil immersion 63 × 1.4 NA Plan Apochromat objective, and a pinhole size of one airy unit. We collected twelve-bit confocal image stacks of 10–15 slices at 0.4 μm Z-step sizes from dual- or triple-labeled cells using the following settings: green channel for detecting Alexa-488, excitation 488 nm Argon laser, emission bandpass filter 505–550 nm; red channel for detecting Alexa-546, excitation 543 nm Helium/Neon laser, emission bandpass filter 560–700 nm (by using the meta monochromator); blue channel for detecting DAPI, excitation 405 nm blue diode laser, and emission bandpass 420–480 nm; blue channel for detecting DRAQ5, excitation 633 nm Helium/Neon laser.

Measurements of fluorescence intensity were taken on a minimum of three confocal stacks per condition, from a single experiment (∼84 cells), using LSM 510 Zeiss software. The background values raised by fluorescent secondary antibodies alone, were subtracted from all samples.

### Immunoprecipitation/Co-immunoprecipitation

To immunoprecipitate PrP^C^ or 37/67 kDa LR, the cells were grown in 100 mm dishes, washed three times with cold PBS and lysed on ice for 20 min in lysis buffer 1 (25 mM Tris-HCl pH 7.5, 150 mM NaCl, 5 mM EDTA, 1% TX-100) with protease inhibitor mixture (leupeptin, antipain, pepstatin, and 1 mM phenylmethylsulfonyl fluoride). The samples were quantified with Bradford assay with Bio-Rad Protein Assay Dye Reagent Concentrate, diluited 1:5 in water; 1.2 mg of proteins were immunoprecipitated, while 80 μg of proteins were kept for totals. Lysates were pre-cleared with protein-A sepharose beads (5 mg/sample) for 30 min at 4 °C and incubated overnight with 1 μg/ml SAF32 or SAF61 anti-PrP antibodies, or with a polyclonal anti-LR 4290 antibody (2 μg/sample) to immunoprecipitate 37/67 kDa LR (both coupled with Protein-A sepharose)^71^. Pellets were washed three times with cold-lysis buffer 1, then boiled with SDS sample buffer 2X (Tris 1M pH 6.8, SDS 10%, glycerol 20% and bromophenole blue and 10% β-mercaptoethanol), loaded on 12% polyacrylamide gels (30% ACRYL/BIS SOL 37.5:1), and revealed by Western blotting with 4290 and/or SAF32 Abs followed by ECL detection (Pierce Euroclone). Densitometric analysis was performed using the free image-processing software Image J.

### Biotinylation assay

GT1 cells grown on dishes were cooled on ice and biotinylated with NHS-LC-Biotin at 4 °C^72^. Cells were lysed for 20 min using buffer 1 (25 mM Tris-HCl (pH 7.5), 150 mM NaCl, 5 mM EDTA, 1% TX-100). Biotinylated cell surface proteins were immunoprecipitated with streptavidin beads (40 μl/sample, Pierce n. 20349). 37/67 kDa LR was specifically immunorevealed with the 4290 pAb and PrP^C^ with SAF32 mAb. In the case of NSC47924 treatment, the cells were first incubated with the inhibitor (or not, control) for indicated different times and then, biotinylated on ice, following the protocol described above.

### Binding of soluble r37LRP to immobilized human recombinant PrP

High binding plates with 96 flat-bottomed wells (Corning, Amsterdam, ND) were coated with 125 ng/well of human recombinant PrP diluted in PBS, or BSA as a negative control, and incubated at 4 °C overnight. After a wash in PBS, residual binding sites were blocked for 1 h at 37 °C with 200 μl of blocking buffer (2% FCS, 1 mg/ml BSA, in PBS). Wells were incubated with 2 μg r37LRP (diluted in PBS, 1 mg/ml BSA), which contained a 6X-His-tag, alone or in presence of NSC47924, for 1 h at 37 °C. Each well was washed three times with wash buffer (0.5% Tween in PBS). Penta-His HRP conjugate (1:500) (Qiagen, Hilden, Germany) was added for 2 h at room temperature. After washing, substrate solution was added and absorbance was detected at 490 nm on an ELISA plate reader (Bio-Rad). Binding was determined by subtracting background absorbance (BSA wells).

### Statistical analysis

Statistical significance of samples against untreated cells was determined by One-way analysis of Variance (ANOVA), followed by the Dunnett’s test. Each value represents the mean ± SEM of at least three independent experiments performed in triplicate (*^,§^*P* < 0.05).

## Additional Information

**How to cite this article**: Sarnataro, D. *et al.* The 37/67kDa laminin receptor (LR) inhibitor, NSC47924, affects 37/67kDa LR cell surface localization and interaction with the cellular prion protein. *Sci. Rep.* **6**, 24457; doi: 10.1038/srep24457 (2016).

## Supplementary Material

Supplementary Information
