# Supplementary material for: The 37/67kDa laminin receptor (LR) inhibitor, NSC47924, affects 37/67kDa LR cell surface localization and interaction with the cellular prion protein
Source: Sci Rep. 2016 Apr 13;6:24457. doi: 10.1038/srep24457 (PMC4829897; doi:10.1038/srep24457)
Supplement: Supplementary Information [file srep24457-s1.pdf]

Supplementary Information

The 37/67kDa laminin receptor (LR) inhibitor, NSC47924, affects 37/67kDa LR cell surface localization and interaction with the cellular prion protein

Daniela Sarnataro[1,2,*], Anna Pepe[1], Gennaro Altamura[3], Imma De Simone[1], Ada Pesapane[4], Lucio Nitsch[1], Nunzia Montuori[4], Antonio Lavecchia[5], and Chiara Zurzolo[6]

[1]Department of Molecular Medicine and Medical Biotechnologies, University of Naples "Federico II", 80131, Naples, Italy; [2]Ceinge-Biotecnologie Avanzate scarl, 80145, Naples, Italy; [3]Department of Veterinary Medicine and Animal Productions, University of Naples “Federico II”, 80137, Naples, Italy; [4]Department of Translational Medical Sciences, University of Naples "Federico II", Naples, Italy; [5]Department of Pharmacy, "Drug Discovery" Laboratory, University of Naples "Federico II", 80131 Naples, Italy; [6]Institut Pasteur, Unité de Trafic Membranaire et Pathogénèse, 75724 Paris CEDEX 15, France.

Figure legends:

Figure S1. 37/67kDa LR does not localize in intracellular compartments. GT1 cells grown on coverslips were fixed, permeabilized with TX-100 0.1% for 10 min and incubated with anti-37/67kDa LR 5004 pAb and with primary antibodies against different markers of intracellular compartments (KDEL, Golgin, EEA1 early endosomes antigen 1). The cells were then stained with anti-mouse and anti-rabbit secondary antibody conjugated with Alexa-488 and Alexa-546. Lysotracker red was used to label lysosomes for 1 hour at 37°C and TfrAlexa-488 to label recycling endosomes for 45 min in vivo before fixation and confocal imaging. Scale bar, 10 µm.

Figure S2. 37/67kDa LR and PrP^C Co-IP in GT1 cells. GT1 cells were grown on 150 mm dishes, lysed and 37/67kDa LR was immunoprecipitated using anti-37/67kDa LR 4290 pAb. PrP^C was revealed in the immunoprecipitate by western blotting by anti-PrP SAF32 antibody. The membrane was stripped and blotted with 4290 pAb to confirm the occurrence of the immunoprecipitation. L: input, IP: immunoprecipitated, SN: 1/10 supernatant of immunoprecipitated proteins, B: protein-A beads alone.

Figure S3. 37/67kDa LR and PrP^C Co-IP in HEK-293 cells. HEK-293 cells were grown and treated as in Fig. 2a.

Figure S4. The inhibitor NSC47924 interferes with cell surface localization of 37/67kDa LR. GT1 cells were grown on coverslips and, after treatment with NSC47924 (or not, control-), were fixed and processed under non-permeabilized conditions for indirect immunofluorescence by labelling 37/67kDa LR (red) and PrP^C (green) with respective primary and secondary fluorescent antibodies. Nuclei were stained with DAPI. Scale bar, 10 μm.

Figure S5. NSC47924 induces accumulation of PrP^C and degradation of 37/67kDa LR in live cells. (a) GT1 cells grown in 60 mm dishes were treated (30 min, 180 min) or not (-) with NSC47924. They were then scraped in lysis buffer and 80μg of total proteins were subjected to SDS-PAGE. 37/67kDa LR and PrP^C were revealed by Western blotting on nitrocellulose and hybridization with a 4290 pAb and SAF32 mAb, respectively. β-tubulin was carried out as loading control. Arrow likely points to 37/67kDa LR degradation fragment. (b) Bands corresponding to 37/67kDa LR and PrP^C were quantified imposing as 100% the amount of both proteins in control conditions, without NSC47924 (-) (N=3, *P<0.05). (c) GT1 cells were treated as in (a) with the exception that here NH4Cl (20 mM, 2 days) was added (+) or not (control) to the culture medium. 37/67kDa LR was revealed by Western blotting on nitrocellulose and hybridization with a 4290 pAb. Note the absence of 37/67kDa LR fragment in NH4Cl panel (arrow). (d) 37/67kDa LR before (-) and after (+) 2 days NH4Cl treatment, was quantified imposing as 100% the amount of 37/67kDa LR without NSC47924 (-) (N=3, *P<0.05). Since the 37/67kDa LR fragment generates

only after 180 min NSC47924 treatment, it was quantified, separately from 37/67kDa LR, imposing as 100% the amount of fragment under 180' NSC47924 (control) but without (-) NH_4Cl.

Figure S6. 37/67kDa LR fragment, generated by NSC47924 treatment, does not reach the cell surface. GT1 cell surface proteins were biotinylated at 4°C in control conditions (without inhibitor, -) or after 180 min of treatment with NSC47924 at 37°C, and were recovered from cell lysates by immunoprecipitation with streptavidin-beads. Total (80 μg of total cell lysates) and cell surface proteins (IP from streptavidin beads), were loaded on gel and processed for SDS-PAGE and ECL. 37/67kDa LR was immunodetected by blotting with 4290 pAb. β-tubulin was carried out as loading control. Note the presence 37/67kDa LR fragment in the total cell lysates and its absence on the cell surface (arrow).

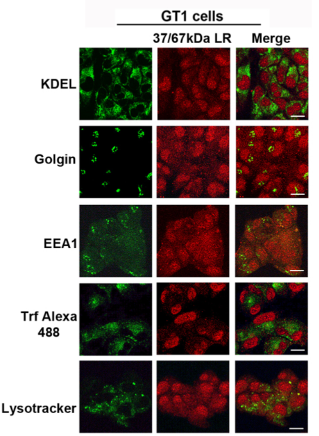
GT1 cells
37/67kDa LR
Merge
KDEL
Golgin
EEA1
Trf Alexa 488
Lysotracker

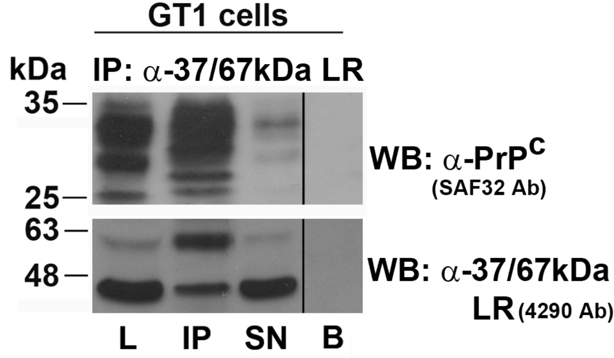

GT1 cells
kDa
IP: α-37/67kDa LR
35
25
63
48
WB: α-PrP^{C}
(SAF32 Ab)
WB: α-37/67kDa
LR (4290 Ab)
L
IP
SN
B

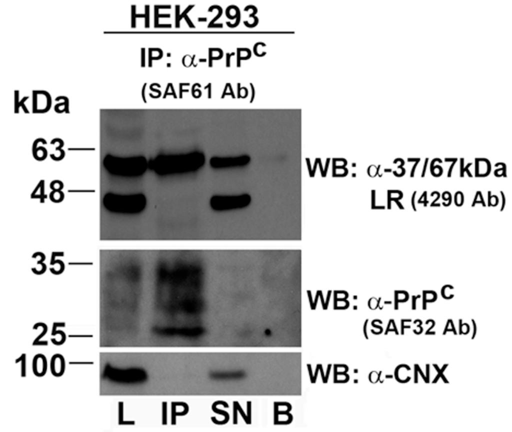
HEK-293
IP: α-PrP^C
(SAF61 Ab)
kDa
63
48
35
25
100
WB: α-37/67kDa
LR (4290 Ab)
WB: α-PrP^C
(SAF32 Ab)
WB: α-CNX
L
IP
SN
B

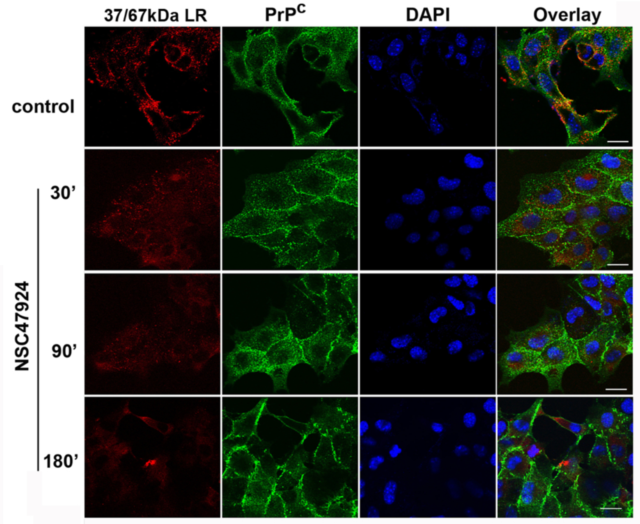

37/67kDa LR
PrPC
DAPI
Overlay
control
NSC47924
30’
90’
180’

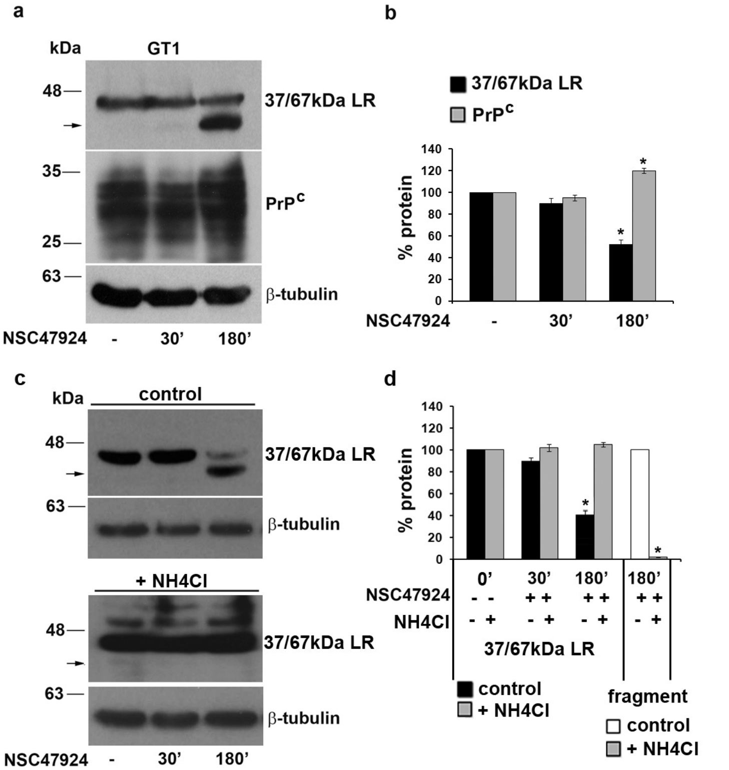

a
kDa
GT1
48
35
25
63
37/67kDa LR
PrP^{c}
β-tubulin
NSC47924
-
30'
180'
b
37/67kDa LR
PrP^{c}
% protein
140
120
100
80
60
40
20
0
*
*
NSC47924
-
30'
180'
c
kDa
control
48
63
37/67kDa LR
β-tubulin
+ NH4Cl
48
63
37/67kDa LR
β-tubulin
NSC47924
-
30'
180'
d
% protein
140
120
100
80
60
40
20
0
*
*
0'
30'
180'
180'
NSC47924
- - + + + + + +
NH4Cl
- + - + - + - +
37/67kDa LR
control
+ NH4Cl
fragment
control
+ NH4Cl

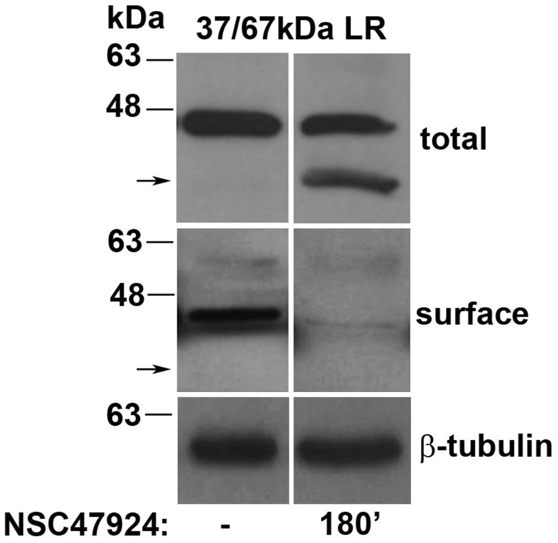
kDa
37/67kDa LR
63
48
total
63
48
surface
63
β-tubulin
NSC47924:
-
180’
